# Remission of type 2 diabetes: A critical appraisal

**DOI:** 10.3389/fendo.2023.1125961

**Published:** 2023-04-03

**Authors:** Michele Ricci, Juan José Mancebo-Sevilla, Lidia Cobos Palacios, Jaime Sanz-Cánovas, Almudena López-Sampalo, Halbert Hernández-Negrin, Miguel Angel Pérez-Velasco, Luis M. Pérez-Belmonte, Maria Rosa Bernal-López, Ricardo Gómez-Huelgas

**Affiliations:** ^1^ Internal Medicine Clinical Management Unit, Hospital Regional Universitario de Málaga, Instituto de Investigación Biomédica de Málaga (IBIMA-Plataforma BIONAND), Malaga, Spain; ^2^ Faculty of Medicine, Universidad de Málaga, Malaga, Spain; ^3^ Servicio de Medicina Interna, Hospital Helicópteros Sanitarios, Marbella, Spain; ^4^ Centro de Investigación Biomédica en Red Fisiopatología de la Obesidad y Nutrición (CIBERobn), Instituto de Salud Carlos III, Madrid, Spain

**Keywords:** type 2 diabetes, remission, no insulin hypoglycemic drugs, weight loss, prevention

## 1 Introduction

There were estimated 463 million people with diabetes in the world in 2019. Epidemiological studies based on population data predict an increase during the next twenty years. By 2045, it is estimated that there will be 700 million people in the world with diabetes (1). A global appeal to the scientific community has been made to create strategies to reduce the prevalence of type 2 diabetes.

Since the introduction of bariatric surgery in the 1990s, a significant improvement in glycemic control began to be observed in patients who underwent this technique. These improvements, which in some cases reached a situation of diabetes remission, were initially attributed to the reduction of caloric intake. In addition, a role of intestinal hormones was hypothesized (2). Later it was demonstrated that the reduction of caloric intake and the consequent weight loss led to a decrease in intrahepatic and intrapancreatic fat, which resulted in an improvement in insulin resistance (3).These findings changed the perception of diabetes from a chronic and progressive disease to a potentially reversible condition. Since then, several studies and consensus documents have been developed to define the concept of remission in type 2 diabetes.

At the same time, hypoglycemic drugs have improved greatly over recent years. The availability of multiple treatment options is leading to important changes in type 2 diabetes management. Traditional diabetes management focused exclusively on glycemic control has been replaced by broader management that includes diabetes complications and comorbidities such a kidney disease, cardiovascular risk, obesity and heart failure.

Through this article we aim to perform a narrative review of the literature on the different strategies studied to achieve remission of type 2 diabetes mellitus, analysing their strengths and limitations. This critical appraisal cannot ignore the advances achieved in the pharmacological management of diabetes and its significance in the prevention of cardiovascular risk as well as the personalized and holistic approach of the diabetic patient.

## 2 Definition of remission in type 2 diabetes

A systematic review of the literature on the topic has identified nearly 100 different definitions of remission in type 2 diabetes (4). This has created a heterogeneity in the studies so far published affecting comparability and reproducibility of the results.

To overcome this issue, an international expert group convened by the American Diabetes Association has recently proposed a definition of remission in type 2 diabetes based on glycated hemoglobin (HbA1c). They define remission as “HbA1c <6,5% (48 mmol/mol) measured at least 3 months after cessation of glucose-lowering pharmacotherapy (at least 6 months after starting a lifestyle intervention)” (5). This document recognises three different interventions through which remission can be achieved: bariatric surgery, pharmacotherapy and lifestyle.

While promoting standardisation, this definition however has a few limitations. Firstly, it brings together under the concept of remission three very different interventions whose results, both from a pathophysiological and clinical point of view, do not seem to be comparable.

In addition, the definition of remission is based on glycosylated hemoglobin levels. This parameter has been chosen because of the high level of reproducibility guaranteed by international standards (6). Nevertheless, it restricts the boundaries of diabetes to an exclusively glucocentric conception without considering the importance of weight loss and other metabolic and cardiovascular changes typical of these patients.

This definition seems to go in the opposite direction to a broader and newer view of type 2 diabetes that has shifted the focus from strict glycemic control to prevention of cardiovascular risk and obesity-associated complications. Indeed, glycosylated hemoglobin levels below 6.5% do not rule out the possibility of developing typical diabetic complications and, as it has been widely demonstrated, cardiovascular risk begins to increase before this cut-off (7, 8).

Considering all these aspects, there is a risk that the label of a “patient in remission for diabetes” may generate a false sense of security in the patient. This may in turn lead to an early abandonment of the necessary follow-up and consequently to inadequate control of the other cardiovascular risk factors. Finally, the new definition of remission is based on expert opinion according to the current diagnostic criteria of T2D. In order to reduce the risk of recurrence, a lower cut-off of HbA1c or using criteria derived from continuous glucose monitoring (CGM) systems, such as time in range (TIR), may be necessary to evaluate diabetes remission (9).The consensus of 2021 states that CGM-derived data can be used, but only the estimated HbA1c (eA1c) level is presented among the values. The data could be considered a novel metric, such as time in range (TIR), in the evaluation of remission.

## 3 Metabolic surgery and remission in type 2 diabetes

Since its introduction in 1990, different techniques to promote weight loss have been developed, including gastric banding, vertical banded gastroplasty, sleeve gastrectomy, Roux-en-Y gastric bypass and biliopancreatic diversion with/without duodenal switching (10).

As a criterium to access the intervention, the US National Institute of Health (NIH) refers to the following parameters: BMI of 40 or greater or BMI of 35 or greater with an obesity-related health condition. Currently we have ample scientific evidence that has demonstrated the efficacy of this measure in achieving and maintaining long-term remission of diabetes.

The effectiveness of this treatment rests not only in promoting significant long-term weight loss.

These intervention determine a dramatically improvement in glycemic control with a reduction in hypoglycemic medication and insulin requirements, leading to remission of diabetes in up to 60-80% of cases in the short term (11, 12).

The most interesting aspect is that remission of diabetes usually occurs very early after surgery (2-3 days to 2-3 weeks) when weight loss has not yet occurred. Several pathophysiological mechanisms have been proposed to explain this improvement. Initially, it has been hypothesised that anatomical changes and the consequent hormonal changes brought about by surgery may play a role. In particular, changes associated with bile acid metabolism, microbiota and the decrease in plasma branched-chain amino acids have been studied (13–16). However, more recent studies have compared the metabolic benefits of dietary intervention alone versus surgery without finding significant differences (17, 18). These studies appear to demonstrate how reduced caloric intake and subsequent weight loss act independently in determining an improvement in glycaemic homeostasis, as measured by different parameters (area under the glycaemic curve, hepatic and muscular insulin sensitivity).

The probability of maintaining long-term remission depends on several factors such as the type of surgery performed, the patient’s ability to make lifestyle changes, the presence of comorbidities and the ß-cell pancreatic reserve.

For instance, the diabetes remission rate is more than twice as high with Roux-en-Y gastric bypass (RYGB) than with laparoscopic adjustable gastric banding, even when adjusted for weight loss (19).

Beyond glycemic control, a recent meta-analysis has studied the long-term effects of bariatric surgery versus conventional treatment in the reduction of cardiovascular events. The results of this study, which included 4 RCT and 6 cohort studies, have shown a significant decrease in macrovascular complications (RR = 0.43, 95%CI = 0.27-0.70), myocardial infarction (RR = 0.40, 95%CI = 0.26-0.61) and cardiovascular events (HR = 0.52, 95%CI = 0.39-0.71) in patients undergoing bariatric surgery versus conservative treatment (20). Moreover, surgery has shown a beneficial effect on the control of hypertension, dyslipidemia and obstructive sleep apnea (21).

Bariatric surgery represents an effective intervention to achieve diabetes remission. Despite its long application, a more extensive understanding of the mechanisms that mediate the improvement of metabolic homeostasis remains to be defined. As a final remark on bariatric surgery, it should be noted that this intervention is aimed at a population with very high cardiovascular risk and with the typical comorbidities of obese patients.

## 4 Lifestyle intervention and remission in type 2 diabetes

Lifestyle modification, including caloric restriction in patients with overweight or obesity and promotion of physical exercise, is the cornerstone of the diabetes therapy. The possibility of reversing alterations in glycemic metabolism through a specific dietary regimen was first assessed in 1976 (22). Since then, numerous studies have attempted to define the type of dietary regimen, its efficacy in glycemic control and its long-term sustainability.

Among those, a retrospective analysis of Look AHEAD study published in 2012, where 5145 patients were randomized to receive intensive lifestyle modification with a low-calorie diet and physical exercise versus conventional diabetes management (23, 24). The intervention group received a low-calorie diet (1200-1800 Kcal/d) and physical exercise (175 minutes per week of moderate to intense activity). The intervention group had a higher rate of diabetes remissions with prevalence of 11.5% (95% CI, 10.1-12.8%) during the first year, and 7.3% (95% CI, 6.2-8.4%) at the fourth year, compared to 2.0% in the conventional treatment group.

Furthermore, in the intervention group there was an improvement in glycemic control (HbA1c) and in most cardiovascular risk factors, such as blood pressure, HDL-cholesterol and triglycerides, but not in LDL-cholesterol, which did not change between the groups.

However, the study suffered an early interruption (9.5 years) because the primary outcomes, which were focused on the reduction of cardiovascular events (acute myocardial infarction, stroke or hospitalization due to angina pectoris) were not achieved.

It should be noted that, compared to the studies performed in the context of bariatric surgery, the population included in this study had very different clinical characteristics. In this study, healthier-than-expected individuals were included, making it more difficult to achieve a benefit in terms of prevention.

Since then, several studies have been conducted assessing the efficacy of different dietary regimens and lifestyle modification strategies on glycemic control. An original study published in 2011 demonstrated the efficacy of caloric intake restriction in determining an improvement in insulin resistance and beta cell functionality. In this study, 11 diabetic patients were subjected to a low-calorie diet (600 kcal/day) for 8 weeks. In addition to the normalisation of plasma glucose levels, a significant decrease in hepatic basal glucose production, an increase in hepatic and peripheral insulin sensitivity and a decrease in hepatic and pancreatic fat content were observed (3). Despite the small sample size, this study was very important in beginning to elucidate the pathophysiological mechanisms underlying the remission of diabetes achieved with a dietary regimen.

Today we know that the very-low-calorie diet (VLCD), a diet that contains 800 kcal per day, achieves the most significant weight loss effect in diabetic patients (25).In this way, a study called the DiRECT clinical trial was conducted with a sample of 306 patients. The intervention consisted of withdrawal of hypoglycemic and antihypertensive drugs, total diet replacement (825–853 kcal per day formula diet for 12–20 weeks), stepped food reintroduction (2–8 weeks), and then structured support for weight-loss maintenance. The control group received an optimized standard treatment. After 24 months of follow-up in the control group, remission of diabetes was observed in 36% and this percentage increased to 86% in patients in whom a weight loss of 15 kg or more was achieved (26). In this study, it was shown that the greater the weight loss the higher remission in diabetes rate.

However, in contrast to the better results in terms of remission compared to those obtained in the Look-AHEAD study, no comparative statistical analysis of cardiovascular outcomes was performed, although a higher number of adverse events were recorded in the control group compared to the intervention group.

In the DIADEM-I study, a similar intervention was performed, subjecting a population of patients with recently diagnosed type 2 diabetes (diabetes duration lower than 3 years) to a low-calorie diet for 12 weeks and a 12-month follow-up (27). The results were compared with a control group that received standard care. At 12 months, 61% of patients in the intervention group had achieved diabetes remission compared to 12% of patients in the control group. In addition, the intervention group showed an improvement in some cardiovascular risk factors such as a decrease in blood pressure and an improvement in the lipid profile. These studies show how a nutritional intervention can be an effective strategy to achieve remission of diabetes, but at the same time a significant and sustained weight loss is necessary. Given that weight loss and maintenance are key to maintaining remission of T2DM and considering that the majority of studies based on life style modification show that weight recovery is the rule in the long-run (ref), a certain risk of diabetes relapse exists in these cases (9). Finally, as with drugs, all interventions on the management of diabetes should necessarily include the repercussions in terms of cardiovascular events among the primary outcomes.

## 5 Hypoglycemic drugs and remission of type 2 diabetes

The possibility of achieving remission of type 2 diabetes through hypoglycemic medication represents the most controversial aspect of this definition. Indeed, the very concept of remission envisages the withdrawal of all types of antidiabetic treatment.

In recent years, we have witnessed great advances in hypoglycemic medication with the diffusion of drugs whose effect has demonstrated a wide range of beneficial effects beyond simple metabolic control and at the same time with an optimal safety profile. Withdrawal of this medication could mean losing the opportunity to prevent the classic complications of type 2 diabetes and the reduction of cardiovascular risk.

The first studies that have investigated the possibility of achieving remission of diabetes with medical treatment have been carried out with therapeutic regimens based on insulin treatment. These studies have been conducted in patients with newly diagnosed diabetes who have undergone intensive insulin treatment with continuous subcutaneous insulin infusion (CSII) for 2-3 weeks) (28–30). After treatment, patients have been followed up not only for short-term glycemic control but also for ß-cell recovery through the homeostasis assessment model (HOMA-B) and other parameters such as measurement of C-peptide, area under the curve (AUC) of insulin and C-peptide or acute insulin response (AIR).

Beyond the quantitative aspects, which are difficult to assess both because of the small sample size and the type of analysis performed, these studies demonstrated how short-term intensive insulin therapy can lead to improved beta-cell function through the reversion of glucotoxicity and a decrease in insulin resistance resulting in improved glycaemic control.

More recently, studies have been carried out with combined therapeutic schemes including insulin and oral hypoglycemic agents. The two most important studies published on this subject are the REMIT-sita and REMIT-dapa studies (31, 32). The objective of both studies was to demonstrate a greater efficacy in achieving diabetes remission and long-term maintenance of an intensive treatment regimen and hygienic-dietary measures compared to traditional management in patients with recent onset type 2 diabetes. In the REMIT-sita study, the intervention arm received combined treatment with sitagliptin, metformin and glargine. On the other hand, in the REMIT-dapa study the treatment was composed of dapaglifozin, metformin and glargine.

Both studies were performed in a population whose characteristics were based on evidence extrapolated from studies performed exclusively with dietary measures, with patients with a short duration of diabetes, baseline HbA1c levels <7.5% and in the absence of comorbidities or diabetic complications. This type of population is the one in which a higher success rate in achieving diabetes remission has been demonstrated. In both studies there was no statistically significant difference between the intervention and control groups.

The GLP1-RA and most recently tirzepatide, a dual agonist of GLP1 and GIP, have shown a very important effect on weight reduction (in order of 10-15%) and therefore may consider potentially agents with a great capacity for diabetes remission in a less aggressive way than surgery, early intensive insulin therapy and VLCD (33, 34). Tirzepatide resulted in a remission rate of diabetes of 66% to 81%, depending on the drug dose over 52 weeks (35). In addition, as demonstrated in the effects of bariatric surgery, the possibility of acting on hormone secretion at intestinal level is of fundamental importance to improve glycemic control. Finally, it is worth mentioning the good safety profile of these drugs, with a low risk of hypoglycemia, with the most frequent adverse effect being the appearance of gastrointestinal minor events (36). However, long-term studies are necessary to define the efficacy of these new drugs for diabetes remission. Furthermore, considering its high cost, may be necessary a selective approach of these therapies for those patients who may achieve higher benefits.

## 6 Conclusions

In the coming years we will witness a significant increase in the prevalence of diabetes in the world population. This increase could be accompanied by a true epidemic of cardiometabolic pathologies with important public health consequences, as well as a significant increase in health care costs.

It is of fundamental importance to implement common strategies at the international level focused not only on the prevention of diabetes but also on the comorbidities and complications most frequently associated with it.

Bariatric surgery represents the most effective measure to achieve and maintain long-term remission of diabetes. In addition, it has demonstrated an effect in the prevention of cardiovascular disease and in general in the control of cardiovascular risk factors. However, it is a measure that has very specific clinical indications considering the associated risks and high costs. Indeed, the surgery is an invasive procedure that can cause serious acute complications, including death (37). For these reasons, it can be applied to a small portion of the diabetic population and is not a measure applicable on a large scale.

The evidence associated with diet and lifestyle interventions demonstrates the efficacy of these measures in achieving diabetes remission status. However, it raises questions about the possibility of large-scale implementation and the long-term duration of their beneficial effects. Furthermore, regarding the efficacy of these measures in terms of cardiovascular risk prevention, it is expected that the beneficial effects on cardiovascular risk factors will lead to a reduction in events and associated mortality. A retrospective analysis of patients included in the DiRECT study has shown a decrease in risk score after the intervention, but no direct evidence of a decrease in mortality and cardiovascular events is available (38).

The studies carried out to date on the possibility of achieving remission of diabetes through a specific therapeutic scheme have not been conclusive. The main limitation of this strategy is the high risk of patient relapse, especially if it is not accompanied by lifestyle changes, close patient follow-up and, more generally, adequate control of other cardiovascular risk factors.

However, the evidence from metabolic surgery and VLCD have identified several factors associated with diabetes remission. Patients with scarce ß-cell function, long-duration of T2D and severe diseases (elevated HbA1c or use of multiple antidiabetic drugs and, particularly, insulin therapy) have lower chance of remission. Timing of intervention is another crucial point to achieve diabetes remission, and early intervention in newly diagnosed T2D can obtain the best results (39).

Taking into account this data, it is possible to identify the patient profile that could benefit from a therapeutic scheme aimed at diabetes remission. The patient should meet the following criteria:

- recent diagnosis of diabetes
- presence of an adequate pancreatic reserve
- low previous need for hypoglycemic treatment
- low baseline glycated hemoglobin levels

However, the opportunity of suspending these treatments should be evaluated. The pleiotropic properties of the new hypoglycemic drugs and their significance in the comorbidities of diabetes and in the prevention of cardiovascular risk question the appropriateness of withdrawing this medication (40, 41).

Weight loss and maintenance are key to maintain diabetes remission and prevent relapse (26).

The possibility of discontinuing antidiabetic medication should be carefully assessed taking into account the patient’s clinical situation, ruling out all patients with renal or cardiac comorbidities or at high cardiovascular risk.

Finally, the importance of a shared decision-making process with the patient should be emphasized. The need for patient commitment to weight control and the high risk of recurrence are fundamental issues to be discussed with the patient In this regard, the Canadian Diabetes Society has proposed a checklist as a standardized information model to encourage full and effective communication by medical staff and, consequently, informed decision making by the patient (42).

## Author contributions

MR, RG-H, and MB-L designed the research study and writing, review and editing the manuscript; LC-P, AL-S, HH-N, and JM-S provide help to write, original graft preparation; RG-H and MB-L get funding for this study. All authors contributed to editorial changes in the manuscript. All authors contributed to the article and approved the submitted version.

## References

[1] Cho NH Shaw JE Karuranga S Huang Y da Rocha Fernandes JD Ohlrogge AW. IDF diabetes atlas: Global estimates of diabetes prevalence for 2017 and projections for 2045. Diabetes Res Clin Pract (2018) 138:271–81. doi: 10.1016/j.diabres.2018.02.023 29496507

[2] Pories WJ Swanson MS MacDonald KG Long SB Morris PG Brown BM. Who would have thought it? an operation proves to be the most effective therapy for adult-onset diabetes mellitus. Ann Surg (1995) 222(3):332–9. doi: 10.1097/00000658-199509000-00011 PMC1234815 7677463

[3] Lim EL Hollingsworth KG Aribisala BS Chen MJ Mathers JC Taylor R. Reversal of type 2 diabetes: Normalisation of beta cell function in association with decreased pancreas and liver triacylglycerol. Diabetologia (2011) 54(10):2506–14. doi: 10.1007/s00125-011-2204-7 PMC3168743 21656330

[4] Captieux M Prigge R Wild S Guthrie B. Defining remission of type 2 diabetes in research studies: A systematic scoping review. PloS Med (2020) 17(10):e1003396. doi: 10.1371/journal.pmed.1003396 33112845 PMC7592769

[5] Riddle MC Cefalu WT Evans PH Gerstein HC Nauck MA Oh WK. Consensus report: Definition and interpretation of remission in type 2 diabetes. Diabetologia (2021) 64(11):2359–66. doi: 10.1007/s00125-021-05542-z 34458934

[6] Jeppsson J-O Kobold U Barr J Finke A Hoelzel W Hoshino T. Approved IFCC reference method for the measurement of HbA1c in human blood. Clin Chem Lab Med (2002) 40(1):78–89. doi: 10.1515/CCLM.2002.016 11916276

[7] Selvin E Steffes MW Zhu H Matsushita K Wagenknecht L Pankow J. Glycated hemoglobin, diabetes, and cardiovascular risk in nondiabetic adults. N Engl J Med (2010) 362(9):800–11. doi: 10.1056/NEJMoa0908359 PMC2872990 20200384

[8] Selvin E Ning Y Steffes MW Bash LD Klein R Wong TY. Glycated hemoglobin and the risk of kidney disease and retinopathy in adults with and without diabetes. Diabetes (2011) 60(1):298–305. doi: 10.2337/db10-1198 20978092 PMC3012185

[9] Kim J Kwon H-S. Not control but conquest: Strategies for the remission of type 2 diabetes mellitus. Diabetes Metab J (2022) 46(2):165–80. doi: 10.4093/dmj.2021.0377 PMC8987695 35385632

[10] Elder KA Wolfe BM. Bariatric surgery: A review of procedures and outcomes. Gastroenterology (2007) 132(6):2253–71. doi: 10.1053/j.gastro.2007.03.057 17498516

[11] Rubino F Gagner M. Potential of surgery for curing type 2 diabetes mellitus. Ann Surg (2002) 236(5):554–9. doi: 10.1097/00000658-200211000-00003 PMC1422611 12409659

[12] Cohen R Caravatto PP Correa JL Noujaim P Petry TZ Salles JE. Glycemic control after stomach-sparing duodenal-jejunal bypass surgery in diabetic patients with low body mass index. Surg Obes Relat Dis Off J Am Soc Bariatr Surg (2012) 8(4):375–80. doi: 10.1016/j.soard.2012.01.017 22410638

[13] Patti M-E Houten SM Bianco AC Bernier R Larsen PR Holst JJ. Serum bile acids are higher in humans with prior gastric bypass: Potential contribution to improved glucose and lipid metabolism. Obes (Silver Spring) (2009) 17(9):1671–7. doi: 10.1038/oby.2009.102 PMC4683159 19360006

[14] Furet J-P Kong L-C Tap J Poitou C Basdevant A Bouillot J-L. Differential adaptation of human gut microbiota to bariatric surgery-induced weight loss: Links with metabolic and low-grade inflammation markers. Diabetes (2010) 59(12):3049–57. doi: 10.2337/db10-0253 PMC2992765 20876719

[15] Graessler J Qin Y Zhong H Zhang J Licinio J Wong M-L. Metagenomic sequencing of the human gut microbiome before and after bariatric surgery in obese patients with type 2 diabetes: Correlation with inflammatory and metabolic parameters. Pharmacogenomics J (2013) 13(6):514–22. doi: 10.1038/tpj.2012.43 23032991

[16] Bradley D Conte C Mittendorfer B Eagon JC Varela JE Fabbrini E. Gastric bypass and banding equally improve insulin sensitivity and β cell function. J Clin Invest (2012) 122(12):4667–74. doi: 10.1172/JCI64895 PMC3512168 23187122

[17] Yoshino M Kayser BD Yoshino J Stein RI Reeds D Eagon JC. Effects of diet versus gastric bypass on metabolic function in diabetes. N Engl J Med (2020) 383(8):721–32. doi: 10.1056/NEJMoa2003697 PMC7456610 32813948

[18] Lingvay I Guth E Islam A Livingston E. Rapid improvement in diabetes after gastric bypass surgery: Is it the diet or surgery? Diabetes Care (2013) 36(9):2741–7. doi: 10.2337/dc12-2316 PMC3747905 23530013

[19] Purnell JQ Selzer F Wahed AS Pender J Pories W Pomp A. Type 2 diabetes remission rates after laparoscopic gastric bypass and gastric banding: Results of the longitudinal assessment of bariatric surgery study. Diabetes Care (2016) 39(7):1101–7. doi: 10.2337/dc15-2138 PMC4915561 27289123

[20] Yan G Wang J Zhang J Gao K Zhao Q Xu X. Long-term outcomes of macrovascular diseases and metabolic indicators of bariatric surgery for severe obesity type 2 diabetes patients with a meta-analysis. PloS One (2019) 14(12):e0224828. doi: 10.1371/journal.pone.0224828 31794559 PMC6890174

[21] Buchwald H Avidor Y Braunwald E Jensen MD Pories W Fahrbach K. Bariatric SurgeryA systematic review and meta-analysis. JAMA (2004) 292(14):1724–37. doi: 10.1001/jama.292.14.1724 15479938

[22] Bistrian BR Blackburn GL Flatt JP Sizer J Scrimshaw NS Sherman M. Nitrogen metabolism and insulin requirements in obese diabetic adults on a protein-sparing modified fast. Diabetes. (1976) 25(6):494–504. doi: 10.2337/diab.25.6.494 1278601

[23] Wing RR Bolin P Brancati FL Bray GA Clark JM Coday M. Cardiovascular effects of intensive lifestyle intervention in type 2 diabetes. N Engl J Med (2013) 369(2):145–54. doi: 10.1056/NEJMoa1212914 PMC3791615 23796131

[24] Gregg EW Chen H Wagenknecht LE Clark JM Delahanty LM Bantle J. Association of an intensive lifestyle intervention with remission of type 2 diabetes. JAMA. (2012) 308(23):2489–96. doi: 10.1001/jama.2012.67929 PMC4771522 23288372

[25] Franz MJ Boucher JL Rutten-Ramos S VanWormer JJ. Lifestyle weight-loss intervention outcomes in overweight and obese adults with type 2 diabetes: a systematic review and meta-analysis of randomized clinical trials. J Acad Nutr Diet. (2015) 115(9):1447–63. doi: 10.1016/j.jand.2015.02.031 25935570

[26] Lean MEJ Leslie WS Barnes AC Brosnahan N Thom G McCombie L. Durability of a primary care-led weight-management intervention for remission of type 2 diabetes: 2-year results of the DiRECT open-label, cluster-randomised trial. Lancet Diabetes Endocrinol (2019) 7(5):344–55. doi: 10.1016/S2213-8587(19)30068-3 30852132

[27] Taheri S Chagoury O Zaghloul H Elhadad S Ahmed SH Omar O. Diabetes intervention accentuating diet and enhancing metabolism (DIADEM-i): arandomised controlled trial to examine the impact of an intensive lifestyle intervention consisting of a low-energy diet and physical activity on body weight and metabolism in. Trials (2018) 19(1):284. doi: 10.1136/bmjopen-2020-041386 29784059 PMC5963071

[28] Ilkova H Glaser B Tunçkale A Bagriaçik N Cerasi E. Induction of long-term glycemic control in newly diagnosed type 2 diabetic patients by transient intensive insulin treatment. Diabetes Care (1997) 20(9):1353–6. doi: 10.2337/diacare.20.9.1353 9283777

[29] Chen A Huang Z Wan X Deng W Wu J Li L. Attitudes toward diabetes affect maintenance of drug-free remission in patients with newly diagnosed type 2 diabetes after short-term continuous subcutaneous insulin infusion treatment. Diabetes Care (2012) 35(3):474–81. doi: 10.2337/dc11-1638 PMC3322723 22228747

[30] Li Y Xu W Liao Z Yao B Chen X Huang Z. Induction of long-term glycemic control in newly diagnosed type 2 diabetic patients is associated with improvement of beta-cell function. Diabetes Care (2004) 27(11):2597–602. doi: 10.2337/diacare.27.11.2597 15504992

[31] McInnes N Hall S Hramiak I Sigal RJ Goldenberg R Gupta N. Remission of type 2 diabetes following a short-term intensive intervention with insulin glargine, sitagliptin, and metformin: Results of an open-label randomized parallel-design trial. Diabetes Care (2022) 45(1):178–85. doi: 10.2337/dc21-0278 34728531

[32] McInnes N Hall S Sultan F Aronson R Hramiak I Harris S. Remission of type 2 diabetes following a short-term intervention with insulin glargine, metformin, and dapagliflozin. J Clin Endocrinol Metab (2020) 105(8):2532–40. doi: 10.1210/clinem/dgaa248 32403130

[33] Wilding JPH Batterham RL Calanna S Davies M Van Gaal LF Lingvay I. Once-weekly semaglutide in adults with overweight or obesity. N Engl J Med (2021) 384(11):989–1002. doi: 10.1056/NEJMoa2032183 33567185

[34] Frías JP Davies MJ Rosenstock J Pérez Manghi FC Fernández Landó L Bergman BK. Tirzepatide versus semaglutide once weekly in patients with type 2 diabetes. N Engl J Med (2021) 385(6):503–15. doi: 10.1056/NEJMoa2107519 34170647

[35] Del Prato S Kahn SE Pavo I Weerakkody GJ Yang Z Doupis J. Tirzepatide versus insulin glargine in type 2 diabetes and increased cardiovascular risk (SURPASS-4): A randomised, open-label, parallel-group, multicentre, phase 3 trial. Lancet (London England). (2021) 398(10313):1811–24. doi: 10.1016/S0140-6736(21)02188-7 34672967

[36] Smits MM Van Raalte DH. Safety of semaglutide. Front Endocrinol (Lausanne). (2021) 12:645563. doi: 10.3389/fendo.2021.645563 PMC8294388 34305810

[37] Morino M Toppino M Forestieri P Angrisani L Allaix ME Scopinaro N. Mortality after bariatric surgery: Analysis of 13,871 morbidly obese patients from a national registry. Ann Surg (2007) 246(6):1002–9. doi: 10.1097/SLA.0b013e31815c404e 18043102

[38] Jesuthasan A Zhyzhneuskaya S Peters C Barnes AC Hollingsworth KG Sattar N. Sex differences in intraorgan fat levels and hepatic lipid metabolism: implications for cardiovascular health and remission of type 2 diabetes after dietary weight loss. Diabetologia. (2022) 65(1):226–33. doi: 10.1007/s00125-021-05583-4 PMC8660759 34657182

[39] Matthews DR Paldánius PM Proot P Chiang Y Stumvoll M Del Prato S. Glycaemic durability of an early combination therapy with vildagliptin and metformin versus sequential metformin monotherapy in newly diagnosed type 2 diabetes (VERIFY): a 5-year, multicentre, randomised, double-blind trial. Lancet (London England). (2019) 394(10208):1519–29. doi: 10.1016/S0140-6736(19)32131-2 31542292

[40] Kristensen SL Rørth R Jhund PS Docherty KF Sattar N Preiss D. Cardiovascular, mortality, and kidney outcomes with GLP-1 receptor agonists in patients with type 2 diabetes: A systematic review and meta-analysis of cardiovascular outcome trials. Lancet Diabetes Endocrinol (2019) 7(10):776–85. doi: 10.1016/S2213-8587(19)30249-9 31422062

[41] Husain M Birkenfeld AL Donsmark M Dungan K Eliaschewitz FG Franco DR. Oral semaglutide and cardiovascular outcomes in patients with type 2 diabetes. N Engl J Med (2019) 381(9):841–51. doi: 10.1056/NEJMoa1901118 31185157

[42] Jin S Bajaj HS Brazeau A-S Champagne J MacDonald B Mackay D. Remission of type 2 diabetes: Users guide. Can J Diabetes (2022) 46(8):762–74. doi: 10.1016/j.jcjd.2022.10.005 36567080

